# Human-AI Collaboration in Healthcare: A Scoping Review

**DOI:** 10.1038/s41746-026-02918-6

**Published:** 2026-06-20

**Authors:** Joshua Strong, Harry Rogers, Emma Sun, Anna Louise Todsen, Jody Ede, Cherry Lumley, Nick Yeung, Helen Higham, J. Alison Noble

**Affiliations:** 1 Institute of Biomedical Engineering, University of Oxford, Oxford, UK; 2 Department of Experimental Psychology, University of Oxford, Oxford, UK; 3 Department of Health and Care Professions, University of Exeter, Exeter, UK; 4 Oxford University Hospital NHS Foundation Trust, Oxford, UK; 5 Nuffield Department of Clinical Neurosciences, University of Oxford, Oxford, UK; 6 Nuffield Department of Anaesthesia, Oxford University Hospitals NHS Foundation Trust, Oxford, UK

**Keywords:** Business and industry, Health care, Medical research, Scientific community

## Abstract

Artificial intelligence is increasingly embedded in clinical pathways, making effective human-AI collaboration (HAIC) a practical and policy priority in healthcare. We conducted a scoping review of empirical studies of HAIC in healthcare published from January 2015 to October 2025, using Joanna Briggs Institute methodology and PRISMA-ScR reporting. Of 17,463 records identified, 140 studies were included. Evidence was concentrated in diagnostic interpretation, with fewer studies in screening and triage, therapeutic decision-making, and administrative workflows. Effectiveness was defined inconsistently across task contexts and was usually assessed using short-term task-level metrics rather than patient or system outcomes. Most studies, particularly in diagnostic interpretation, reported benefits for human-AI teams. These depended on task fit, workflow integration, training, and appropriately calibrated trust. Ethical and governance issues, including accountability and patient safety, were often discussed but rarely evaluated empirically. These findings provide a foundation for more task-specific, longitudinal, and governance-aware evaluation of HAIC in healthcare.

## Introduction

Artificial intelligence (AI) is increasingly embedded across clinical pathways, ranging from image interpretation and triage to documentation and care coordination. However, in decision-critical healthcare settings, the value of AI deployment cannot be assessed solely by whether systems autonomously outperform clinicians on isolated tasks. Clinical practice is characterised by safety constraints, professional accountability, and context-dependent judgement, motivating approaches in which humans and AI systems collaborate under meaningful human oversight. In this review, human-AI collaboration (HAIC) refers broadly to configurations in which humans and AI systems jointly contribute to a healthcare task. This includes both judgement-centred tasks, in which AI may inform interpretation or decision-making, and review-and-sign-off workflows, in which AI generates candidate outputs that require human validation, improvement where needed, and final sign-off.

Regulatory and policy developments have reinforced HAIC as an operational expectation for clinical AI deployment. For instance, the World Health Organisation’s guidance on the ethics and governance of large multimodal models emphasises lifecycle risk management, transparency, and the involvement of qualified professionals in deployment and use^1^. The European Union’s AI Act requires that high-risk AI systems be designed and deployed with human-oversight measures that enable effective monitoring and intervention to minimise risks to health, safety, and fundamental rights^2^. In the U.S., the Food and Drug Administration (FDA) has published *Transparency for Machine Learning-Enabled Medical Devices: Guiding Principles*, in which Principle 7 explicitly focuses on the performance of the human-AI team^3^. These frameworks place a focus on the consideration of how performance is embedded within clinical workflows, including questions of responsibility, control, and decision authority in practice.

HAIC encompasses a range of configurations in which clinicians (licenced health care professionals involved in patient care decisions) and AI systems coordinate who does *what* and *when*. For conceptual orientation, these can be grouped into three broad modes: AI-as-assistant or second-reader support, triage or task-allocation workflows, and propose-and-verify workflows. In some settings, AI supports clinicians through prioritisation, decision support, or structured summarisation; others are designed to manage task allocation, including approaches in which AI defers selected cases to human experts when additional judgement is expected to add value through their clinical expertise. Such systems are designed to induce complementarity across the task space rather than to replace human expertise. Ultimately, these systems shape performance: they influence trust calibration, cognitive workload, situational awareness, and the distribution of responsibility, all of which determine whether an AI system is used appropriately and safely in a real-world clinical environment.

Despite rapid growth in this area, the evidence supporting the use of HAIC in healthcare remains fragmented. Terminology varies across disciplines (e.g., human-AI teaming, hybrid intelligence, human-in-the-loop decision support), study designs range from laboratory simulations to small-scale clinical deployments, and outcomes span performance, efficiency, usability, reliance and override behaviour, perceived trustworthiness, and safety-relevant events. Recent quantitative syntheses have begun to characterise team-level effects, for example, a reliability-analysis of 52 empirical clinical studies found that AI can augment clinician performance, but that full complementarity is uncommon and effects vary by teaming mode and clinician expertise^4^. However, existing syntheses are either broader than healthcare or do not reflect recent advances in interactive and generative AI systems, limiting their usefulness for clinicians, health-system leaders, and designers^5^. Consequently, there is a lack of clarity on how HAIC is conceptualised, implemented, and evaluated across healthcare contexts. A scoping review is therefore appropriate because the field is conceptually broad and methodologically heterogeneous, and our aim is to map the range of definitions, study designs, outcomes, and evidence gaps rather than estimate a single pooled effect.

At the same time, documented risks including automation bias, over-reliance on AI, inequitable performance across patient groups, highlight several socio-technical factors that can determine the effectiveness of HAIC systems^6,7^. Outcomes depend not only on model performance but also on interface design, workflow integration, organisational readiness, training, and ongoing monitoring, including the ability to detect drift, misuse, or shifts in clinician reliance. These risks strengthen the need to systematically map what has been studied, how HAIC has been evaluated, and where key gaps remain for safe, equitable, and accountable deployment.

We therefore conducted a scoping review of the literature on HAIC in healthcare from 1 January 2015 to 27 October 2025, to address three research questions: (1) how is effectiveness defined and evaluated across clinical task contexts? (2) which technical, human, and organisational determinants shape successful human-AI collaboration? and (3) what ethical, safety, and governance requirements are reported for accountable HAIC? The review aims to inform future research, system design, and the governance of HAIC in healthcare.

## Results

**Fig. 1:**
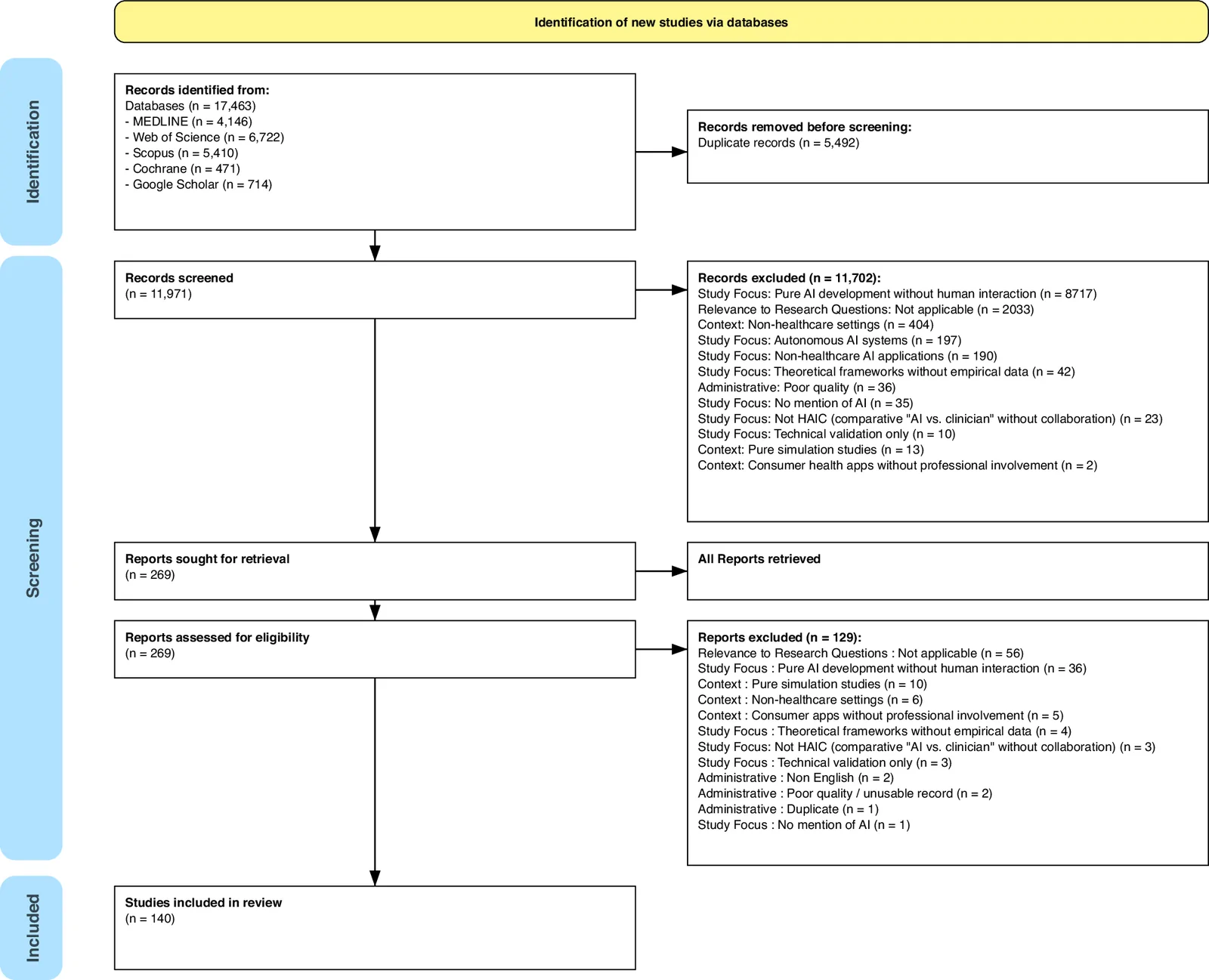
The PRISMA-ScR flow diagram of the study selection. Flow diagram showing record identification, duplicate removal, title/abstract screening, full-text eligibility assessment with reasons for exclusion, and final inclusion of studies in the scoping review. Created with open-source software PRISMA2020^19^.

The initial search across 5 databases yielded 17,463 results. After removing duplicates, and screening by title and abstract, 269 papers were retrieved for full-text assessment against the predefined inclusion and exclusion criteria. After full-text screening, 140 studies were included in this review (Fig. 1).

### RQ1: How is effectiveness defined and evaluated across clinical task contexts?

Evaluation Approaches and Metrics

**Fig. 2:**
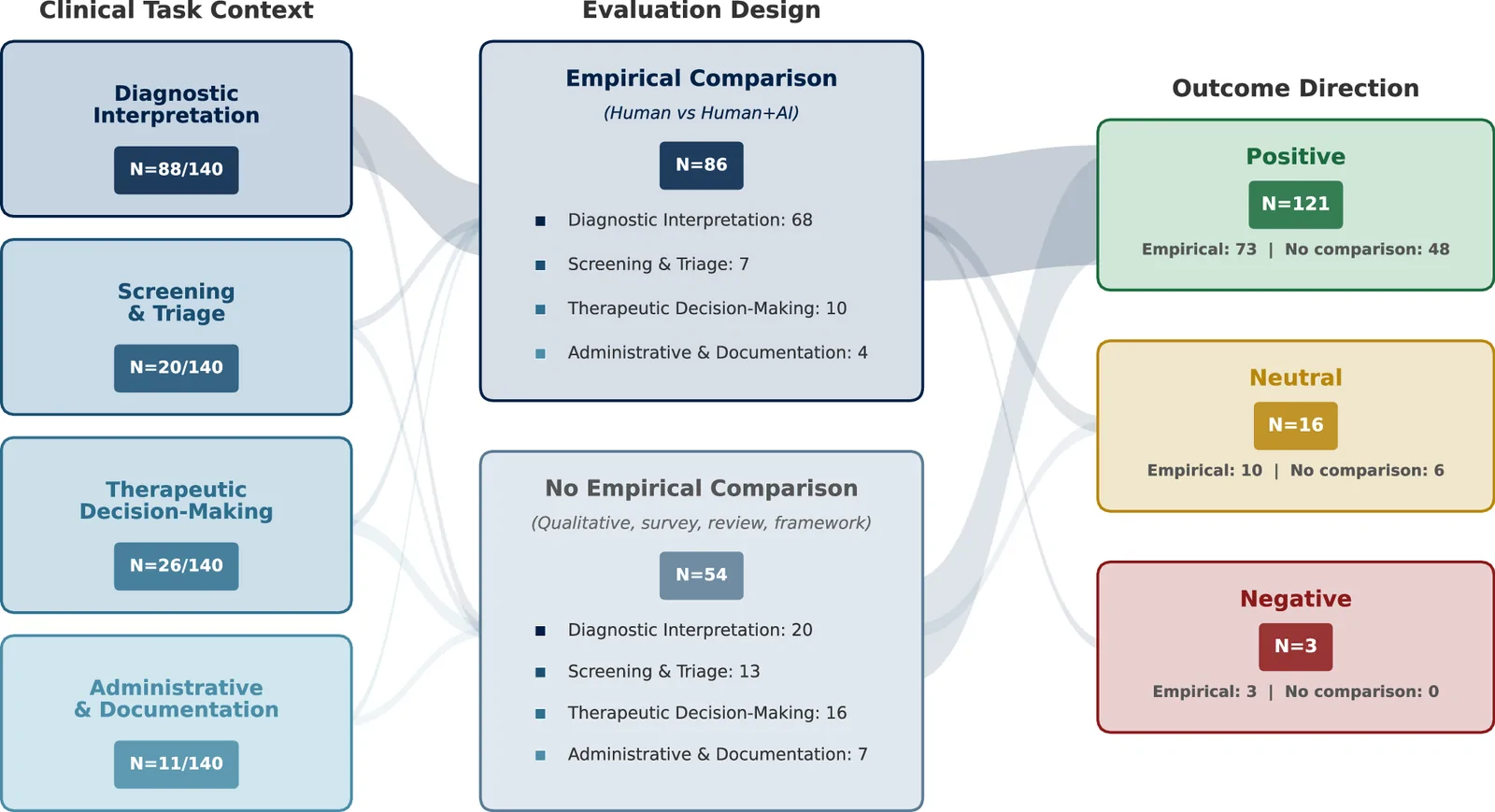
Distribution of included studies by clinical task context, evaluation design, and outcome direction. Alluvial plot showing how included studies were mapped from clinical task context to evaluation design and reported outcome direction. Clinical task categories were not mutually exclusive, as studies could be coded to more than one context. Evaluation design distinguishes studies with a direct empirical comparison of human-only versus human-AI performance from studies without such a comparison. Outcome direction was coded descriptively as positive, neutral/mixed, or negative according to each study’s reported outcomes. These counts should not be interpreted as pooled estimates of HAIC effectiveness because tasks, study designs, and outcome measures were heterogenous.

Evaluation strategies for HAIC in healthcare are methodologically diverse but typically compare human-AI teams against human-only decisions, with some also benchmarking against AI-only performance, to determine the effectiveness of the collaboration. We present evidence organised by clinical task context, moving from more bounded administrative and documentation workflows to diagnostic interpretation, screening and triage, and therapeutic decision-making and planning. Across these contexts, the outcomes used to evaluate effectiveness differed: administrative and documentation studies emphasised workflow fit, acceptability, and time savings; diagnostic interpretation studies most often used task-level performance metrics; screening and triage studies emphasised pathway speed, prioritisation, and resource use; and therapeutic decision-making studies more often combined process, human-factor, and downstream clinical outcomes. We summarise the distribution of included papers by clinical task context, evaluation design, and reported outcome direction in Fig. 2.

### Administrative and Documentation Workflows

Administrative and documentation workflows (*N* = 11/140) represented the smallest subset of included studies. Across all 11 studies in this set, AI produced suggestions (e.g., candidate codes, drafted notes/transcripts, structured histories, or verification outputs) that required clinician/coder/pharmacist review and final sign-off. Effectiveness was frequently operationalised through human-factor and implementation outcomes: 9/11 included qualitative enquiry (e.g., ethnography, interviews, observations, or open-text feedback) to characterise perceived usefulness, workflow fit, and acceptability, and 3/11 reported quantitative experience measures such as Likert ratings, or trust scales. Objective efficiency outcomes were reported in 3/11 (documentation time reduction, coding time, or reaction time), while 1/11 captured perceived time savings via clinician questionnaires. Where an external “correct” answer was available, 4/11 evaluated output quality using task-level performance or agreement metrics (e.g., accuracy/F1/AUC for classification, number of HCC codes, or kappa/ICC for scoring). For example, long-term fieldwork with clinical documentation integrity specialists described AI-enabled computer-assisted coding as a “rough draft” that specialists iteratively validate and correct under full responsibility^8^. A primary-care scribe implementation similarly evaluated effectiveness as reduced clinician documentation time alongside stakeholder feedback on rapport and engagement^9^.

### Diagnostic Interpretation

Diagnostic tasks accounted for the largest share of studies (*N* = 88/140). These studies often involved tasks with reference standards and commonly used comparative reader-style evaluations. Across the full review, 86/140 studies included empirical human-only versus human-AI comparisons; within diagnostic interpretation, 67/88 did so. Of these 67 comparative diagnostic studies, 42 used multi-reader or within-subject designs in which clinicians interpreted cases with and without AI, and 36 also reported AI-only performance^10–15^. These experiments typically randomise case order or use crossover designs with washout periods and compare conditions on standard diagnostic and timing metrics. Across the full review, the remaining 54/140 studies did not include empirical human-only versus human-AI comparisons and instead used qualitative, survey, or other non-comparative empirical designs.

Many diagnostic studies evaluated configurations in which AI reduced the search burden, such as via first-pass scanning, region highlighting, or prioritisation, while clinicians retained responsibility for final decision-making. For example, in a neonatal EEG study, an AI-driven attention mechanism modulated sonified playback to compress long recordings ( ~ 1 hour into ~5 s) while achieving seizure-presence detection accuracy comparable to experienced neurophysiologists interpreting the full EEG^16^. In radiology, studies report that AI assistance improved residents’ hip-fracture detection on X-rays and acted like a second reader for non-radiology clinicians interpreting emergency chest radiographs, including findings that are clinically relevant and can be easily overlooked^10,11,17,18^.

Among diagnostic studies with quantifiable outcomes, accuracy was the most frequently reported primary metric (*N* = 39/73), followed by sensitivity (*N* = 12/73), time and efficiency measures (*N* = 9/73), AUC (*N* = 7/73), and inter-rater agreement metrics (*N* = 6/73). Many studies report multiple metrics; thus our counts reflect the primary outcome measure. For instance, in capsule endoscopy, prostate MRI, and emergency neuroimaging, studies have reported that AI assistance increased sensitivity or AUC for clinicians without large reduction in specificity, although the magnitude and consistency of benefit varied by task and reader experience^5–7,19^.

### Screening and Triage Workflows

Screening and triage studies (*N* = 20/140) have used similar modalities to diagnostic interpretation. Relative to diagnostic interpretation, these studies more often evaluated whether AI helped route attention and prioritise urgent cases within high-volume pathways, and accordingly emphasised workflow^20^, time^18,21,22^, and efficiency metrics^23^ alongside detection rates. Time-to-decision and pathway metrics are central in emergency and acute care implementations. For instance, AI-assisted stroke triage software was evaluated through door-in-door-out times and interhospital transfer intervals, while aneurysm detection tools and AI-filtered imaging workflows were assessed by reading time, report turnaround, and time saved per case^24,25^.

Beyond simple time measurements, these studies evaluate screening workflows in terms of system-level implementation, feasibility, and resource use. In a sepsis study, an EHR-integrated AI triggered best-practice alerts, and the evaluation emphasised real-world user interaction and process metrics (for example, alert-to-action ratio, screening completion, and protocol initiation), alongside quality outcomes such as a risk-adjusted sepsis mortality metric^26^. In a cardiomyopathy trial, AI-guided screening used point-of-care predictions from a digital stethoscope and asynchronous AI-ECG predictions, with positive AI screens requiring echocardiographic confirmation for primary endpoint determination; effectiveness was evaluated primarily by the rate of identified left-ventricular systolic dysfunction versus routine obstetric care (clinician-directed evaluation, with echocardiography ordered as clinically indicated) rather than clinician time^27^. In a mobile low-dose computed tomography program, clinicians reviewed scans with an AI nodule detection system and completed structured reports while screening a large underserved rural cohort, and feasibility and cost-effectiveness were assessed using detection/diagnosis rates and economic metrics including cost-effective ratios (and the early detection cost index)^23^.

### Therapeutic Decision-Making and Planning

Therapeutic decision-making and planning studies (*N* = 26/140) more often evaluated effectiveness using measures downstream of prediction accuracy, and measures were often combined within the same study. Four of 26 defined effectiveness using patient-level clinical outcomes in real care or procedural pathways: an asthma decision-support RCT used exacerbations and asthma control status plus healthcare utilisation/costs (and electronic health record review time)^28^, a sepsis CDS implementation reported alert-driven process adherence (e.g., alert-to-action and protocol initiation) alongside a risk-adjusted sepsis mortality metric^26^, AI-assisted quantitative coronary angiography reported procedure time, contrast volume, and in-hospital/cardiac mortality^29^, and augmented-reality preoperative planning reported operative time, blood loss, resection completeness, complications, and hospital stay^30^. Six of 26 included explicit time/throughput measures (e.g., interaction time, appointment length, EHR review time, procedure/operative time)^28,31^. Six of 26 operationalised effectiveness as decision/action change or decision safety, including counterfactual prescribing-rule simulations (antibiotic use and overprescribing)^32^, clinician acceptance/override behaviours and dose changes (with stopping/help-request rates)^33^, and treatment-plan acceptance or action measures (including alert-to-action ratios)^26,34^. Eight of 26 reported performance-style metrics for the AI, clinician, or team (used either to characterise intermediate outputs or decision quality where a single “correct” answer was limited) such as AUC/accuracy, F1 agreement, intraclass correlation, or segmentation similarity (e.g., Dice/HD)^32,35,36^. Finally, 16 of 26 treated human factors and implementation outcomes as part of effectiveness, quantifying perceived usefulness, workload/workflow impact, trust or weight-of-advice, and/or implementation constructs (acceptability/appropriateness/feasibility or adoption drivers), with qualitative components used in 16 of 26 studies to elicit clinician/patient preferences, barriers, and workflow implications^26,35,37–39^.

### RQ2: What technical human, and organisational determinants shape successful human-AI collaboration?

Across the 140 included reports, direction findings were reported more frequently than neutral or negative findings. Approximately 86% (*N* = 121/140) of evaluations reported positive-direction findings on study-defined outcomes, including task performance, efficiency, or user acceptance of human-AI teams compared with human-only practice, with the remaining minority describing neutral or negative effects. This figure should not be interpreted as a pooled estimate of HAIC effectiveness, because included outcomes were heterogeneous, many studies were conducted in controlled settings, and relatively few linked collaboration to patient-relevant, system-level, or calibration-based outcomes. When examined by task context, positive-direction findings were reported in administrative/documentation workflows (9 positive, 1 neutral, 1 negative), diagnostic interpretation (75 positive, 11 neutral, 2 negative), screening and triage (17 positive, 3 neutral, 0 negative), and therapeutic decision-making and planning (23 positive, 3 neutral, 0 negative). Because some studies were coded to more than one task context, these category counts are not mutually exclusive. This indicates that positive-direction findings were observed across task types, but that the largest empirical base remains diagnostic interpretation.

### Technical Performance and Interaction Design

A first cluster of factors relevant to successful collaboration relate to technical performance and interaction design. Of the 87 studies with empirical human vs human + AI comparisons, the large majority reported positive outcomes (*N* = 76/87). Systems that were shown to be accurate, reliable, and complementary to human strengths consistently improved human-AI team performance. Examples included AI tools that increased sensitivity for rib and thoracic pathology on chest radiographs without sacrificing specificity^13^, AI support for intracranial haemorrhage detection that performed best as a second reader alongside radiologists^12^, and assistance for PET/CT lymphoma staging^40^, colonoscopy, and capsule endoscopy^15,21,41^ that raised detection rates or reduced interpretation time. Successful HAIC regarding real-time guidance and automation of routine sub-tasks included accelerating triage of large-vessel occlusion stroke transfers^24^, guiding image acquisition at the point of care^42,43^, or pre-segmenting structures for human review^40,44–46^. Conversely, when AI outputs generated frequent false alarms^25^ or the interface was poorly integrated into clinicians’ workflow^47^, studies reported increased review burden and disrupted task performance^48^. Explainability and uncertainty communication emerged as prominent design features (*N* = 53), but demonstrated both positive and negative effects on HAIC. Many studies (*N* = 38/53) reported that visual or case-based explanations, such as heatmaps localising suspected lesions, example-based reasoning, or confidence scores, improved interpretability and diagnostic accuracy^20,49–53^. However, several experiments (*N* = 22/53) also showed that explanation format matters as some explanation styles increased cognitive load or induced over-reliance when users inferred more certainty from the AI than was warranted^49,52,54–56^.

### The Importance of Trust

**Fig. 3:**
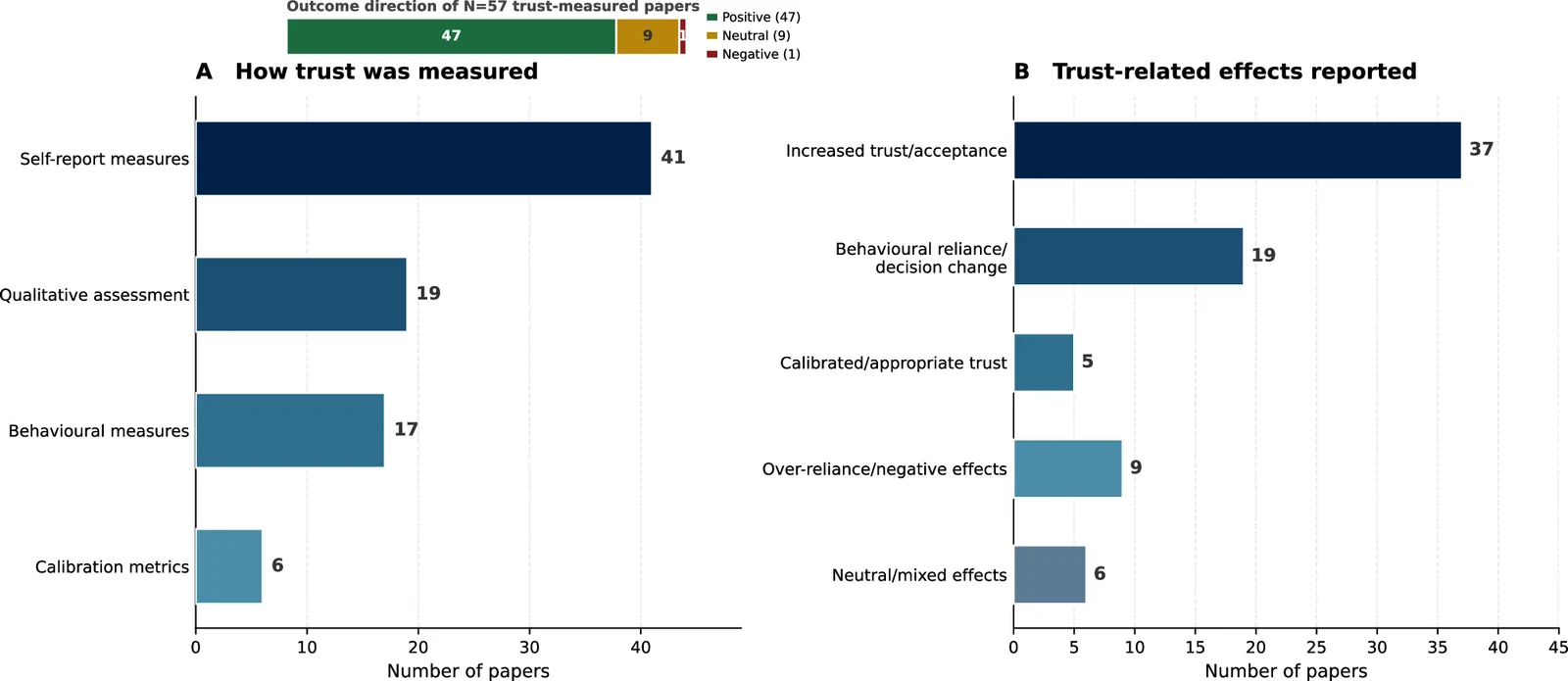
Trust measurement and trust-related findings across included HAIC studies. Among the 57 studies that explicitly measured trust, self-report measures were the most common approach, followed by qualitative assessments, behavioural measures, and calibration metrics. Reported trust-related effects included increased trust or acceptance, observed reliance or decision change, calibrated or appropriate trust, over-reliance or negative trust effects, and neutral or mixed effects. Measurement and effect categories were not mutually exclusive, as studies could use multiple trust measures or report multiple trust-related outcomes. The inset shows the overall direction of trust-related findings across these studies.

Trust is central to HAIC because it shapes whether clinicians choose to engage with AI, when they rely on it, and when they override recommendations. Here, we use trust in the sense of a willingness to accept vulnerability under uncertainty based on expectations about the system’s performance, making it important both as a precondition for uptake and as a determinant of the quality of human-AI decision-making^57–59^. The ways trust was measured and the types of trust-related effects reported across included studies are summarised in Fig. 3.

Across the included studies, approximately 41% (*N* = 57/140) explicitly measured trust. Trust was primarily (*N* = 41/57) measured through self-reported metrics (i.e., structured survey items or scales capturing self-perceived trust, confidence, willingness to use, perceived accuracy, etc), but also by qualitative/contextual means (*N* = 19/57) (i.e., insights from interviews, focus groups, etc plus assessments of explainability methods), observed behavioural actions (*N* = 17/57) (i.e., adoption rate, overriding of AI decisions, decision-changing metrics etc), and a minority of studies (*N* = 6/57) measured trust via calibration metrics (i.e., appropriate trust rate, concordances with gold-standards). Because trust was measured heterogeneously and most often through self-reported or qualitative/contextual approaches, the included literature does not allow robust characterisation of the direction or magnitude of any divergence between stated trust and observed reliance. Accordingly, increases in reported trust or willingness to use HAIC should not be interpreted as evidence of appropriately calibrated reliance.

Outcomes related to trust in HAIC systems demonstrated heterogeneous findings, which we group into five themes: (i) increased trust and adoption; (ii) behavioural reliance and decision change; (iii) calibration and outcome-linked trust; (iv) miscalibration, over-reliance, and negative trust effects; and (v) neutral or mixed effects.

#### Increased trust

This captures studies in which HAIC systems were associated with higher self-reported trust and greater self-reported willingness to use or adopt the system. 28 of 57 studies reported increases in subjective trust, confidence, or willingness to adopt HAIC systems. Common outcomes included increased trust and related constructs such as higher decision confidence, increased willingness to use the system, and improved perceived usefulness. Trust was observed to strengthen when AI systems provided clear explanations, performance feedback, and visual or quantitative outputs.

#### Behavioural reliance and decision change

This group of studies captured an overall positive observable clinician behaviour in response to working with HAIC systems (*N* = 11/57) and demonstrated how trust translated into appropriate decision-making. Studies documented measurable shifts in clinician behaviour including an appropriate reliance on AI, higher acceptance rates, alignment with AI recommendations, reduced override rates, greater advice-taking, fewer second opinions, and direct adjustments to clinical decisions or treatment plans^30,35,36,56–58^.

#### Calibration and performance outcomes

This concerns reported studies where clinician trust was appropriately aligned with AI competence (*N* = 11/57). Studies reported outcomes such as higher diagnostic accuracy, increased agreement with AI when appropriate, improved reliance on AI for correct outputs, and performance improvements across expertise levels^51,60–69^. Trust calibration was influenced by factors such as the relevance and quality of explanations, certainty or confidence signals from the AI, transparency, alignment with clinical reasoning, and system performance feedback. When these when these factors were incorporated in system design, studies demonstrated more appropriate reliance patterns alongside improve human-AI task performance. For example, a clinician study that explicitly varied whether clinical decision support system recommendations were correct versus incorrect found explanation conditions improved overall performance (defined as agreeing with correct recommendations and rejecting incorrect ones) compared with no explanation^63^. Behavioural reliance was sometimes (*N* = 3/11) associated with over-reliance, automation bias, or confirmation bias, particularly when explanations were perceived as highly reassuring or when time pressure influenced decision-making^55,63,70^.

#### Miscalibration and negative trust effects

Trust was not always found to be beneficial. Several studies (*N* = 10/57) described over-reliance, confirmation bias, and automation bias with clinicians sometimes accepting incorrect AI recommendations. This led to reduced diagnostic accuracy, increased severity of automation bias, or poor performance in cases where the AI was wrong^66,71^. Conversely, under-reliance also occurred with clinicians demonstrating low trust, alert dismissal, reluctance to adopt, or increased scrutiny of AI outputs^72,73^. Studies also documented trust collapse after exposure to errors or fallibility where trust declined significantly even when performance remained within expected bounds^33,74^.

#### Neutral or mixed effects

A subset (*N* = 5/57) of studies reported neutral or mixed effects across trust-related measures. Outcomes included no measurable change in confidence or self-reported trust. However, unchanged confidence does not necessarily indicate unchanged trust, as behavioural reliance may vary independently^22,25,37,46,75^. In some cases, clinicians engaged critically with ambiguous outputs rather than displaying either trust or distrust. Two studies found flat reliance patterns despite added explanations or workflow integration^37,75^.

### Organisational Context and Implementation

A subset of *N* = 11/140 papers had implementation focused study designs. Accordingly, organisational determinants should be interpreted as emerging from a smaller and more heterogeneous evidence base than the diagnostic performance literature, although related workflow, feasibility, acceptability, and adoption themes also appeared in studies across other task contexts. Implementation orientation papers stressed that embedding AI models into existing clinical information systems and workflows with minimal disruption, for example through seamless EHR integration or non-intrusive alerts, facilitated adoption and sustained use^9,13,24,38,72,76^. Strategic and cultural aspects of organisations also shaped success. For instance, mixed-methods studies on implementation in hospitals and radiotherapy services discussed the importance of visible leadership, multi-stakeholder collaboration, and the early engagement of clinicians, IT, management, and patients in defining use cases and success criteria^9,38,76,77^. Training and onboarding were repeatedly identified as enabling conditions for successful utilisation of HAIC (*N* = 35/140). Studies that provided targeted training on AI-based tools, explanation formats, and uncertainty information reported better diagnostic performance and more appropriate reliance, particularly among novices: resident doctors^11,17^ and novice operators^42^.

### RQ3: What are the ethical implications for accountability and patient safety?

#### Ethical Implications for Accountability

Ethical implications related to accountability (*N* = 27/140) were repeatedly presented as interaction problems rather than properties of AI models alone. Responsibility, trust, and oversight were discussed in relation to how they are distributed across human-AI teams and their surrounding governance structures. A consistent ethical concern was that opaque or weakly interpretable AI can make it difficult for clinicians to justify, contest, or appropriately calibrate reliance on AI outputs. This created both practical barriers to safe use and downstream challenges when investigating adverse outcomes. Thirteen papers converged on a position where the clinician remains responsible. Even when AI improves performance or efficiency, diagnostic and treatment responsibility was typically framed as staying with the human professional^12,18,41,68,78,79^. This stance was sometimes justified as necessary given current limitations in AI reliability and explainability^16,18,80^. However, two papers also highlighted an emerging tension. While responsibility was retained by clinicians in principle, decision influence was increasingly distributed across vendors, developers, implementers, and workflow designers^81,82^. In this context, accountability risks became blurred in practice, particularly when AI was integrated as a second reader, triage layer, or embedded recommender that shaped attention and thresholds without transparent rationale^83,84^. This responsibility-control mismatch was also reflected in patient-facing work where uncertainty about liability and expectations of disclosure raise ethical pressure for clearer institutional and legal guidance on who is answerable when AI meaningfully contributes to clinical decisions^85^.

#### Patient Safety Risks

Patient safety concerns (*N* = 31/140) were described through two linked mechanisms: the possibility of direct clinical error propagation from incorrect AI recommendations, and interaction-mediated harms such as automation bias, confirmation bias, anchoring, and time-pressure effects that can make clinicians more likely to accept erroneous suggestions or insufficiently scrutinise outputs^33,55,86,87^. These risks were not confined to resident doctors; three studies also suggested differential vulnerability by role and experience. For example, non-specialists and resident doctors may benefit from assistance while also being more prone to uncritical acceptance^11,17^, and deskilling and out-of-the-loop performance problems were raised only sparsely in the included literature and were not systematically evaluated prospectively. In one qualitative ICU study, higher levels of automation may contribute to “out-of-the-loop” performance problems, whereby clinicians become less able to detect and respond to system errors, reinforcing the need to maintain human autonomy, transparency, and meaningful engagement in decision-making^88^.

#### Strategies for Safe and Accountable Design

Across the included studies, mitigation strategies did not treat “human-in-the-loop” oversight as sufficient on its own, but instead described specific design and organisational measures required to ensure safe and accountable human-AI collaboration. The most frequently proposed design strategy was explainability aiming to ensure that AI reasoning, features, or case-based comparisons are accessible to clinicians (*N* = 24/140). Related design strategies included strengthening onboarding and training, so clinicians understand AI capabilities and failure modes (*N* = 14/140), and embedding mechanisms for contestability and override as part of routine use (*N* = 11/140). At the system level, a smaller number of papers proposed governance frameworks specifying roles, escalation pathways, and regulatory requirements (*N* = 6/140), accountability and traceability measures such as documentation of when and how AI influenced decisions (*N* = 6/140), and ongoing monitoring and quality assurance to detect performance drift in deployed settings (*N* = 6/140) Overall, the ethical picture that emerges is that accountability and patient safety are inseparable. Without transparency, traceability, and well-specified human-AI workflows, clinician responsibility can become a default legal position that does not by itself prevent predictable interaction failures that compromise safety^55,63,82,88–91^.

## Discussion

Across 140 empirical studies published between 1 January 2015 and 27 October 2025, research on HAIC in healthcare reports many benefits but is hard to compare across settings. This review makes three main contributions: it shows that HAIC effectiveness is task-dependent rather than a single uniform construct, it identifies trust, workflow integration, and training as recurrent but unevenly evaluated determinants of successful collaboration, and it highlights a persistent gap between governance expectations for human oversight and what empirical studies evaluate. For RQ1, effectiveness was clearly task-dependent, with different task contexts emphasising different outcome frameworks and therefore resisting simple aggregation. For RQ2, most evaluations report better performance, faster work, or higher acceptance with human-AI teams, but these gains depend on how the system fits the task and the workflow: benefits are clearest when AI helps with specific, well-bounded steps (e.g., prioritising cases, highlighting areas, drafting text) and clinicians keep responsibility for the final decision, whereas poor integration or frequent false alarms can add work and disrupt care. However, this conclusion is supported most strongly by diagnostic interpretation, which constituted the largest and most standardised evidence base. The smaller literatures in screening and triage, therapeutic decision-making, and administrative/documentation also more often reported positive than neutral or negative findings, but used more heterogeneous outcome measures and therefore support narrower, more context-specific claims. Trust is central but inconsistently assessed: only 57/140 studies measured it directly, often using self-reported surveys rather than observed behaviour, and studies disagree on whether explanations and confidence displays reliably lead to appropriate use. Accordingly, the overall positive-direction rate is best understood as a descriptive summary of reported study outcomes rather than as a direct estimate of clinical effectiveness, particularly because many endpoints were task-level, implementation, or self-reported measures rather than patient or system outcomes. For RQ3, accountability and patient safety are often discussed, but these issues are rarely built into the main evaluation – so there is a gap between policy expectations for “human oversight” and what studies actually test. The rest of this discussion is organised around the three research questions. We first consider how effectiveness is defined across clinical task contexts and what current evaluations often miss (RQ1), then discuss the technical, human, and organisational determinants of successful collaboration (RQ2), and finally consider the implications for trust, accountability, patient safety, and governance (RQ3).

For RQ1, a primary interpretive finding is that “effectiveness” in HAIC is operationally defined by clinical task context rather than by a single common metric. Across the included literature, this ranged from workflow fit, review burden, and time savings in administrative/documentation tasks, to signal-detection and reading-time metrics in diagnostic interpretation, to pathway prioritisation in screening and triage, and to more mixed process, human-factor, and downstream clinical outcomes in therapeutic decision-making. This has direct methodological implications: future syntheses and trials should avoid aggregating “HAIC effectiveness” indiscriminately across task types, and should instead stratify evidence by the clinical activity being augmented and the task properties that shape collaboration (e.g., context dependence, time criticality, and relational content).

Administrative and documentation workflows warrant separate conceptual treatment from diagnostic interpretation. In the administrative/documentation studies included here, the human role was not to collaborate with AI toward a diagnosis, but to verify, edit where necessary, and authorise AI-generated drafts under retained accountability. These systems therefore sit closer to “propose-and-verify” task allocation than to shared clinical judgement. Their benefits and risks are different: they may improve standardisation or efficiency, but they can also introduce hidden verification work and the possibility of passive checking. Accordingly, these studies were included as one mode of HAIC at the workflow layer, but they should not be treated as directly commensurable with evidence on diagnostic interpretation or therapeutic decision-making. This reinforces our broader point that HAIC effectiveness is task dependent and should not be aggregated indiscriminately across task types.

Several included studies, mainly in diagnostic interpretation and training-related contexts, suggest an emerging pattern in which HAIC may confer larger gains for less experienced clinicians and non-specialists, while more experienced users may gain efficiency through reduced search burden and fatigue. Because expertise was operationalised heterogeneously across studies and expertise-stratified effects were not systematically extracted across all task types, this should be interpreted as a post-hoc interpretive hypothesis rather than a formal review finding. If this pattern holds, in settings where care is often delivered by generalists or non-specialists and specialist backup is limited, HAIC may act as a safety net by reducing missed findings and supporting timely escalation. In training contexts, it may provide scaffolding that accelerates learning and reduces exposure to rare-but-important errors. However, deskilling and role erosion remain plausible but under-studied, particularly when AI shifts clinicians from active interpretation to passive checking. There is also countervailing evidence that less experienced users may be more vulnerable to uncritical acceptance and automation-related harms when AI is wrong. This tension suggests that HAIC design should not focus solely on successful task completion, but also on sustaining and developing clinician expertise over time. Systems should be designed to keep users cognitively engaged, encourage independent reasoning before exposure to AI recommendations, and adapt the level of support to experience and context. Rather than functioning as a fixed, continuously enabled support layer, HAIC tools should help clinicians remain skilled, attentive, and capable of recognising when the system may be wrong.

Despite current evidence that HAIC can improve accuracy and efficiency in controlled settings, there remains a substantial gap regarding impact measured in real-world settings. Many evaluations stop at the decision point (correct diagnosis, time saved, agreement), while few track whether these gains translate into altered management, reduced adverse events, or improved patient-relevant outcomes. This matters because clinical utility is mediated by workflow and organisational response: better detection does not guarantee better outcomes if thresholds, escalation pathways, staffing, or treatment access remain unchanged. Progress therefore requires a shift from predominantly short-term reader studies toward longitudinal, in-situ evaluations that connect collaboration to patient and system endpoints, including failure modes (e.g., missed deterioration, delayed escalation, inappropriate de-escalation) as well as intended benefits.

Efficiency metrics can also obscure the “hidden work” introduced by collaboration. Qualitative and mixed-methods studies show that HAIC often creates new work rather than removing it: clinicians verify outputs, arbitrate disagreements, manage false positives, document rationale, and explain system behaviour to colleagues or patients^8^. These oversight tasks are essential for safety but can erode net time savings and add cognitive load that is not captured by throughput measures alone. At the same time, an under-emphasised value proposition of HAIC is standardisation. Even where AI models do not outperform the best human experts, collaboration can reduce variability across a clinical team by supporting consistency in detection, scoring, and prioritisation. This suggests that a complete account of “efficiency” should include not only speed, but also cognitive effort, coordination burden, and the degree to which collaboration promotes reliable, reproducible decision-making across settings and providers.

For RQ2, reported success was contingent not on AI performance alone, but on alignment between technical design, clinician use, and organisational context. Technically, the included studies suggest collaboration is most likely to be effective when AI supports bounded sub-tasks that complement clinicians (prioritisation, highlighting, draft generation, pre-segmentation) while humans retain integrative judgement and accountability. Explainability and uncertainty communication emerged as important design features, but their effects are nuanced: some formats were shown to improve interpretability and performance, whereas others increase cognitive load or induce over-reliance when users infer more certainty than warranted. A smaller implementation-oriented literature emphasised workflow integration (including EHR integration and non-intrusive alerts), visible leadership, and multi-stakeholder involvement in defining use cases and success criteria. Training and onboarding repeatedly appear as enabling conditions, particularly for novices such as resident doctors, suggesting that “human-in-the-loop” is not achieved simply by placing a clinician at the end of the pipeline; it requires training and ongoing support so clinicians can interpret outputs, calibrate reliance, and intervene appropriately when the system is uncertain or wrong. However, only 11/140 included studies had implementation-focused designs, so these organisational determinants should not be read as having the same evidential weight as the much larger task-performance literature. They are better interpreted as convergent signals from a smaller and more heterogeneous evidence base, consistent with broader clinician-adoption and AI-implementation literature^92^. Dedicated implementation-science and hybrid effectiveness--implementation research is needed to evaluate how HAIC can be integrated, adopted, and sustained in real clinical settings.

For RQ3, the literature suggests that trust, accountability, and patient safety are best understood as interactional properties of HAIC rather than as isolated attributes of AI systems. Yet trust is inconsistently measured: only a minority of studies assess it directly, and many rely on self-reporting metrics even though stated trust can diverge from observed reliance. This matters because reliance can be beneficial when it is calibrated to AI competence, but harmful when it produces automation or confirmation bias. Accordingly, the aim of HAIC design should not be simply to increase trust, but to support appropriately calibrated trust, so that clinicians rely on AI when it is likely to be helpful and question or override it when it is not. A practical implication is that HAIC evaluations should triangulate (i) subjective trust ratings, (ii) behavioural measures such as advice-taking, override, and escalation, and (iii) calibration metrics that link reliance to objective correctness under varying error rates and time pressure.

Ethical and governance papers reinforce the same interaction framing for accountability and safety. While many studies adopt the position where the clinician remains responsible, they also highlight a responsibility-control mismatch: AI systems can shape attention, thresholds, and triage decisions through interface design and workflow integration, even when clinicians retain formal liability. Accordingly, proposed mitigations extend beyond generic “human oversight” to concrete safeguards, including clear communication of uncertainty and intended-use boundaries, standardised presentation of limitations, routine documentation of when AI influenced decisions, and well-defined escalation and override pathways.

In conclusion, HAIC is increasingly positioned as an important mode of deploying AI safely and effectively in healthcare. This scoping review demonstrates that the evidence base is growing rapidly, but remains fragmented across task types, study designs, and conceptualisations of collaboration. Our results suggest that reported benefits of HAIC are most consistently demonstrated in diagnostic interpretation, while smaller literatures in screening and triage, therapeutic decision-making, and administrative/documentation also more often report positive-direction findings, albeit using more heterogeneous outcome measures. Across these contexts, benefits appear most likely when AI is applied to bounded sub-tasks and clinicians retain responsibility for judgement.

We identify three implications for the successful integration of AI in healthcare. First, evaluation must become more task- and context-specific. Effectiveness should not be treated as a single construct across administrative work, diagnostic interpretation, screening and triage, and treatment planning; instead, future studies should assess HAIC using outcome frameworks that reflect the clinical purpose of the task, including not only accuracy and efficiency but also workflow impact, cognitive burden, and downstream patient and system outcomes. Second, successful collaboration requires consideration to human and organisational factors, particularly trust calibration, interface design, workflow integration, and training. Integration requires systems that support clinicians without encouraging over-reliance, passive checking, or deskilling, and that are designed around real clinical environments. Third, accountability and patient safety must be treated as core evaluative concerns and not secondary ethical reflections. Human oversight alone is insufficient unless it is supported by transparency, contestability, traceability, and clear organisational governance over how AI influences decisions in practice.

Our findings suggest that future HAIC research should progress from short-term reader studies and toward longitudinal and in-situ evaluations of how collaboration reshapes clinical work, patient outcomes, and professional responsibility over time, including whether repeated reliance affects clinician skill retention or out-of-the-loop vulnerability. This should include implementation-science and hybrid effectiveness–implementation studies that examine uptake, workflow integration, and sustainment alongside effectiveness. Progress will depend on designing better human-AI systems that are clinically useful, organisationally workable, and ethically governable. More mature evidence base for HAIC should therefore centre on three priorities: task-matched evaluation, human-centred and context-sensitive design, and explicit governance for safe and accountable use. These will be essential if AI is to be integrated intelligently into healthcare in ways that strengthen clinical judgement and patient care.

These findings should be interpreted in light of several limitations. As a scoping review, our aim was to map and synthesise patterns in the literature rather than estimate pooled effects, and we did not conduct a formal risk-of-bias assessment or a formal assessment of publication bias. Our deliberately broad operational definition of HAIC was appropriate to the scoping aim but encompassed diverse collaboration modes, including judgement-centred decision support and propose-and-verify workflows, which increased heterogeneity and may reduce direct comparability across some included study types. We also restricted the review to English-language studies, which may have excluded relevant evidence from non-English-speaking settings. Although we complemented database searching with Google Scholar and reference-list checking, we did not undertake a dedicated search of grey-literature repositories; for a topic with substantial policy and implementation relevance, this may have reduced capture of implementation-focused evaluations and other practice-based evidence not indexed in major bibliographic databases. In addition, the study-level positive-direction rate should be interpreted cautiously because included outcomes were heterogeneous and often comprised task-level, implementation, or self-reported measures rather than patient-relevant or calibration-based outcomes. The evidence base we identified is concentrated in controlled evaluations of diagnostic interpretation and likely over-represents positive findings. Accordingly, broader claims about HAIC are supported most strongly in diagnostic tasks and may understate real-world failure modes in other contexts. We also did not systematically extract expertise-stratified effects across all included studies nor deskilling or longitudinal performance degradation as standalone outcomes; accordingly, observations about a possible “levelling effect” should be read as interpretive rather than definitive, and findings regarding deskilling should be interpreted as research gaps rather than review findings. These limitations point to the need for research that moves beyond isolated task comparisons toward evaluations that are explicitly matched to clinical task properties and workflow constraints. In practice, this means developing task-stratified evaluation frameworks that combine performance with workflow impact, safety-relevant outcomes, and human factors, and then testing these frameworks in longitudinal, in-situ studies that link collaboration to patient and system endpoints rather than decision-point accuracy alone. Similarly, because trust and reliance are central mechanisms through which HAIC can help or harm, future work should measure them using complementary approaches: self-report, observed behaviour, and calibration metrics that relate reliance to objective correctness under realistic error profiles and time pressure.

## Methods

### Protocol and reporting

This scoping review followed a prespecified protocol prospectively registered on the Open Science Framework (OSF; DOI: 10.17605/OSF.IO/N9RYF) and was conducted in line with the Joanna Briggs Institute (JBI) methodology for scoping reviews, with reporting guided by PRISMA-ScR^93^. The registered protocol specified five research questions addressing evaluation methods, clinical tasks contexts, trust, operational success factors, and ethical/governance implications. During manuscript preparation, these were consolidated for reporting into three overarching questions: effectiveness in task context (integrating the original evaluation and task-context questions); determinants of successful collaboration (integrating trust with broader technical, human and organisational success factors); and ethics, safety, accountability, and governance (integrating the original ethics question with governance-related success factors). This consolidation was undertaken because effectiveness metrics were task-dependent, trust overlapped with broader determinants of collaboration, and governance overlapped with accountability and patient-safety analyses. It changed only the reporting structure: the review scope, eligibility criteria, search strategy, data charting, and included data were unchanged, and all extracted data were mapped to the consolidated framework. A completed PRISMA-ScR checklist is provided in Supplementary data A.

### Search strategy and information sources

We searched MEDLINE (via PubMed), Scopus, Web of Science, and the Cochrane Library, and complemented database searching with Google Scholar. Searches targeted English-language records published from 1 January 2015 to 27 October 2025. We consulted a librarian in developing search strings. Search strings combined terms for human-AI collaboration (e.g., “human-AI collaboration”, “human-AI teaming”, “hybrid intelligence”, “human-AI interaction”, “AI assist*”) with healthcare context terms and collaboration/interaction operators (e.g., collab*, team*, interact*, complement*), and were adapted to each database; full strategies are provided in Supplement B. Reference lists of included studies were also checked to identify additional eligible records.

### Eligibility criteria

We applied a PCC (Participants-Concept-Context) framework. Participants were clinicians and other healthcare stakeholders (including patients) engaged in tasks involving collaboration with AI. The concept of interest was human-AI collaboration broadly defined (including “human-AI teaming” and “hybrid intelligence”), where humans and AI systems jointly contribute to a healthcare task. Operationally, this included both decision-support configurations in which AI informed human interpretation or decision-making and propose-and-verify workflows in which AI generated candidate outputs requiring human review, revision where needed, and final sign-off. Studies of fully autonomous AI without human input were excluded. The context was healthcare settings (e.g., hospitals, clinics, community/telehealth) with empirical evaluation or observation. We included empirical primary studies (experimental, observational, qualitative, mixed-methods, pilots/case studies, or validation studies with human users) and excluded purely technical/engineering or simulation-only work without clinical end-users.

### Study selection

Records were deduplicated and screened in Rayyan^94^. Two reviewers independently screened titles/abstracts and full texts, resolving disagreements through discussion and consensus. The selection process is summarised in a PRISMA flow diagram (Fig. 1).

### Data charting

We developed and piloted a structured extraction form based on the protocol and JBI guidance, refined it iteratively during piloting before full charting, and double-charted all included studies. Discrepancies in charted data were resolved through discussion and consensus between reviewers. Extracted items included: study metadata and setting; collaboration model (e.g., AI-as-assistant/second-reader/propose-and-verify/mixed-initiative/deferral); task category/categories, with studies coded to more than one task context where applicable; success factors (technical/human/organisational/system); definitions and measures of trust; evaluation methods and outcome metrics (e.g., diagnostic performance, time/workflow, errors, cost, usability, workload, acceptance/reliance); and ethical/governance topics (e.g., accountability, safety, bias/fairness, privacy/consent, patient and public involvement). The full extraction template and data items are provided in the Supplement.

### Data synthesis

We performed descriptive mapping (counts, distributions, and evidence maps) and thematic synthesis across the review questions; no quantitative meta-analysis was planned. The synthesis used a hybrid deductive-inductive approach: charting domains were structured from the protocol and review questions, while outcome groupings and cross-study themes were refined iteratively during analysis. Given the breadth of the HAIC concept, findings were synthesised by task context and thematic domain rather than treating HAIC as a single homogeneous intervention class. Consistent with scoping review guidance, we did not undertake formal risk-of-bias assessment or grade certainty of evidence, but narratively note important design limitations where relevant. The authors used ChatGPT (OpenAI) to assist with language editing and refinement of manuscript wording. All scientific content, interpretation, and final editorial decisions were made by the authors.

### Patient and public involvement

No patients or members of the public were involved in the design, conduct, reporting, or dissemination plans of this scoping review.

## Supplementary information

Supplementary Information

Supplementary Information

## Data Availability

The data supporting the findings of this review are available within the article and its supplementary materials. Extracted information from the included studies are summarised in themain text, and the standardised Excel extraction sheet used for data collection is provided as a supplementary file.
