# Supplementary material for: Human-AI Collaboration in Healthcare: A Scoping Review
Source: NPJ Digit Med. 2026 Jun 20;9:768. doi: 10.1038/s41746-026-02918-6 (PMC13642898; doi:10.1038/s41746-026-02918-6)
Supplement: Supplementary file 1 [file 41746_2026_2918_MOESM1_ESM.pdf]

Supplementary material

A: PRISMA-ScR checklist

Data 1: PRISMA-ScR checklist

Item	PRISMA-ScR reporting item	Reported?	Location in manuscript / notes
1	Title: Identify the report as a scoping review.	Yes	Title: "Human-AI Collaboration in Healthcare: A Scoping Review".
2	Structured summary in abstract (background, objectives, eligibility, sources, methods, results, conclusions).	Yes	Abstract includes background, objective, methods, date range, key results, and conclusions/implications.
3	Rationale: Describe rationale in context of existing knowledge and why scoping review is appropriate.	Yes	Introduction: rationale, policy context (WHO, EU AI Act, FDA), and need to map a fragmented evidence base.
4	Objectives: Provide explicit objectives/questions (e.g., PCC).	Yes	Introduction: three research questions (RQ1–RQ3). Methods: PCC framework described under Eligibility criteria.
5	Protocol and registration: Indicate protocol exists, where accessed, and registration info.	Yes	Methods, Protocol and reporting: OSF protocol DOI 10.17605/OSF.IO/N9RYF.
6	Eligibility criteria: Specify characteristics of sources (years, language, publication status) and rationale.	Yes	Methods, Eligibility criteria: 1 Jan 2015–27 Oct 2025; English-language; empirical primary studies; exclusions specified.
7	Information sources: Describe all sources (databases, other) and dates covered; date last searched.	Yes	Methods, Search strategy and information sources: MEDLINE (PubMed), Scopus, Web of Science, Cochrane Library, Google Scholar; searches covered publications from 1 Jan 2015 to 27 Oct 2025.
8	Search: Present full electronic search strategy for at least one database.	Provided in Supplement	Methods, Search strategy and information sources; full search strategies provided in Supplementary material B.

Item	PRISMA-ScR reporting item	Reported?	Location in manuscript / notes
9	Selection of sources: State selection process (screening/eligibility).	Yes	Methods, Study selection: deduplication, Rayyan screening, two independent reviewers, disagreements resolved by discussion/consensus.
10	Data charting process: Describe methods (forms, calibration, independent/duplicate).	Yes	Methods, Data charting: piloted structured extraction form; all included studies double-charted; supplementary extraction template referenced.
11	Data items: List/define variables sought and assumptions/simplifications.	Yes	Methods, Data charting: study metadata, collaboration model, task category, trust measures, evaluation methods/metrics, and ethical/governance topics listed.
12	Critical appraisal (if done): Rationale and methods; how used.	N/A	No formal risk-of-bias or critical appraisal undertaken; stated in Methods, Data synthesis.
13	Synthesis of results: Describe methods for summarizing charted data.	Yes	Methods, Data synthesis: descriptive mapping (counts, distributions, evidence maps) and thematic synthesis; no meta-analysis planned.
14	Selection of sources (results): Numbers screened/eligible/included; reasons for exclusions; flow diagram.	Yes	Results and Figure i: PRISMA flow diagram with numbers screened, full-text assessed, and included.
15	Characteristics of sources: For each included source, present characteristics and citations.	Yes	Results provide aggregated characteristics by task context and study examples. A full study-characteristics table is provided in the Supplementary.
16	Critical appraisal within sources (if done): Present appraisal data.	N/A	No critical appraisal performed.
17	Results of individual sources: Present relevant data for each included source.	Yes	Narrative synthesis includes study-level examples, but reporting is mainly aggregated. A study-level extraction is supplied in the Supplementary CSV.
18	Synthesis of results: Summarize charted results in relation to objectives/questions.	Yes	Results structured around RQ1–RQ3 with synthesis across task contexts, determinants of success, and ethics/governance.

Item	PRISMA-ScR reporting item	Reported?	Location in manuscript / notes
19	Summary of evidence: Summarize main results and relevance to stakeholders.	Yes	Discussion summarizes key findings and implications for research, design, implementation, and governance.
20	Limitations: Discuss limitations of the scoping review process.	Yes	Discussion: limitations paragraph notes absence of formal risk-of-bias assessment, concentration in diagnostic studies, and likely over-representation of positive findings.
21	Conclusions: Provide general interpretation and potential implications/next steps.	Yes	Final paragraphs of the Discussion provide overall interpretation and next steps, including task-stratified evaluation, longitudinal in-situ studies, trust measurement, and governance needs.
22	Funding: Describe sources of funding for included evidence (if available) and for the review; role of funders.	Yes	Acknowledgements list EPSRC/NIHR funding and fellowships.

Data 1: A completed PRISMA-ScR checklist containing 20 essential and 2 optional items for our scoping review.

B: Search strategies

Search strategy for MEDLINE (via PubMED) with service provider U.S. National Library of Medicine

("human ai collab*"[All Fields] OR "human ai team*"[All Fields] OR "hybrid intell*"[All Fields] OR "human ai interact*"[All Fields] OR "ai assist*"[All Fields] OR "ai recommendation*"[All fields] OR "Artificial Intelligence"[MeSH Terms]) AND ("delivery of health care"[MeSH Terms] OR "delivery of health care"[All Fields] OR "healthcare"[All Fields] OR "health care"[All Fields]) AND ("collab*"[All Fields] OR "interact*"[All Fields] OR "team*"[All Fields] OR "coop*"[All Fields] OR "co-op*"[All Fields] OR "complement*"[All Fields] OR "Human-machine collaboration"[All Fields] OR "cooperative behavio*r"[MeSH Terms]) AND (2015/01/01:3000/12/31[Date - Publication] AND "English"[Language] AND ("clinical trial"[Publication Type] OR "Review"[Publication Type] OR "journal article"[Publication Type]))

Search strategy for Web of Science:

ALL=("human ai collab*" OR "human ai team*" OR "hybrid intell*" OR "human ai interact*" OR "ai assist*" OR "ai recommendation*" OR "Artificial Intelligence") AND ALL=("delivery of health care" OR "healthcare" OR "health care") AND ALL=(collab* OR interact* OR team* OR coop* OR co-op* OR complement* OR "Human-machine collaboration" OR "cooperative behavio*r") AND LA=(English) AND DT=(Article OR Review) AND PY=(2015-2025)

Search strategy for Scopus:

TITLE-ABS-KEY (("human ai collab*" OR "human ai team*" OR "hybrid intell*" OR "human ai interact*" OR "ai assist*" OR "ai recommendation*" OR "Artificial Intelligence") AND ("delivery of health care" OR "healthcare" OR "health care") AND (collab* OR interact* OR team* OR coop* OR co-op* OR complement* OR "Human-machine collaboration" OR "cooperative behavio*r")) AND PUBYEAR > 2014 AND LANGUAGE (english) AND DOCTYPE (ar OR re)

Search strategy for Cochrane Database of Systematic Reviews:

((human ai collab*) OR (human ai team*) OR (hybrid intell*) OR (human ai interact*) OR (ai assist*) OR (ai recommendation*)) AND ((delivery of health care) OR (health*care)) AND ((collab*) OR (interact*) OR (team*) OR (coop*) OR (co-op*) OR (complement*) OR (Human-machine collaboration) OR (cooperative behavio*r))

Search strategy for Google Scholar via Publish or Perish:
Search 1:
Title words: "AI-assisted" OR "AI-augmented"
Keywords: (healthcare OR clinical) (clinician OR physician OR nurse OR doctor OR patient) ("user study" OR "reader study" OR trial OR evaluation) -robotics -manufacturing -education
Years: 2015-2025

Search 2:
Title words: "human-AI collaboration" OR "human AI collaboration" OR "human-AI collaborative"
Keywords: (healthcare OR clinical) (clinician OR physician OR nurse OR doctor OR patient) ("user study" OR "reader study" OR trial OR evaluation) -robotics -manufacturing -education
Years: 2015-2025

Search 3:
Title words: "human-AI teaming" OR "human AI teaming"
Keywords: (healthcare OR clinical) (clinician OR physician OR nurse OR doctor OR patient) ("user study" OR "reader study" OR trial OR evaluation) -robotics -manufacturing -education
Years: 2015-2025

Search 4:
Title words: "hybrid intelligence" OR "hybrid-intelligence"
Keywords: (healthcare OR clinical) (clinician OR physician OR nurse OR doctor OR patient) ("user study" OR "reader study" OR trial OR evaluation) -robotics -manufacturing -education
Years: 2015-2025
